# The importance of early life touch for psychosocial and moral development

**DOI:** 10.1186/s41155-019-0129-0

**Published:** 2019-08-02

**Authors:** Darcia Narvaez, Lijuan Wang, Alison Cheng, Tracy R. Gleason, Ryan Woodbury, Angela Kurth, Jennifer Burke Lefever

**Affiliations:** 1 Department of Psychology, University of Notre Dame, Notre Dame, IN 46556 USA; 2 Department of Psychology, Wellesley College, Wellesley, MA 02481-8203 USA

**Keywords:** Touch, Affection, Corporal punishment, Morality, Wellbeing, Maternal attitudes

## Abstract

One of the primary means of communicating with a baby is through touch. Nurturing physical touch promotes healthy physiological development in social mammals, including humans. Physiology influences wellbeing and psychosocial functioning. The purpose of this paper is to explore the connections among early life positive and negative touch and wellbeing and sociomoral development. In study 1, mothers of preschoolers (*n* = 156) reported their attitudes toward positive/negative touch and on their children’s wellbeing and sociomoral outcomes, illustrating moderate to strong positive correlations between positive touch attitudes and children’s sociomoral capacities and orientations and negative correlations with psychopathology. In study 2, we used an existing longitudinal dataset, with at-risk mothers (*n* = 682) and their children to test touch effects on moral capacities and social behaviors in early life. Results demonstrated moderate to strong relationships between positive/negative touch and concurrent child behavioral regulation and positive correlations between low corporal punishment and child sociomoral outcomes. In a third study with adults (*n* = 607), we found significant mediation processes connecting retrospective reports of childhood touch to adult moral orientation through attachment security, mental health, and moral capacities. In general across studies, more affectionate touch and less punishing touch were positively associated with wellbeing and development of moral capacities and engaged moral orientation.

“Touch is one of the central experiences of an infant, whether rodent, primate, or human. We readily think of stressors as consisting of various unpleasant things that can be done to an organism. Sometimes a stressor can be the failure to provide something to an organism, and *the absence of* touch is seemingly one of the most marked of developmental stressors that we can suffer” (Sapolsky, 1994, p. 92).

Children learn to be human through touch. Touch is the earliest form of sensory experience for a developing human being. Prenatally, the womb provides a constant sensation of being held. Postnatally, babies expect a similar level of feeling connected through the “in arms” care of mother and others. Experience of touch in early life influences the neurobiological development of multiple systems in mammals. However, the implications of touch for complex behaviors that depend upon these neurobiological systems, such as morality and sociality, are less clear.

## Purpose of research

The purpose of the paper is to address whether early touch relates to later social and moral wellbeing. We approach the question using several methods: a cross-sectional study of mothers and pre-school-aged children, a longitudinal study of first-time mothers and their children followed from pregnancy to age 3 years, and a retrospective study of adults’ early childhood experiences and current socio-moral functioning. While previous research (Field, 1985, 1995, 2010) has examined the implications of early touch experience for physiological and emotional health, we investigated linkages between touch experience and outcomes that reflect psychological health and sociomoral development.

Psychological interest in the effects of early life touch can be traced back to the mid-twentieth century, when systematic experiments showed that lack of affectionate touch resulted in extensive negative outcomes in young mammals. For example, Harlow (1958) found that rhesus monkeys, isolated only from touch, developed aberrant social skills. James Prescott (1996) was the first to identify a sensory deficit disorder among children who received little positive touch but plenty of negative touch through punishment. This touch experience was linked to later addictions and violence, and Prescott postulated that such experience communicated “badness” to the child. Likewise, Spitz (1945) noted the depression that human babies experienced (“hospitalism”) in institutions where they received custodial care (bottle feeding and changing) but little affectionate touch.

### Early touch and physiological development serves as a base for socio-moral behavior

Integrating ethological and anthropological research, Bowlby (1951) pointed out the importance of touch for young humans, describing the importance of bonding and attachment with caregivers—processes that require responsive touch in early life. These findings connecting touch to myriad physiological and social outcomes suggest that touch might have a role to play in regulatory systems of social development. More recently, experiments with mammals demonstrate maternal touch’s profound physiological effects in the short term on growth (Schanberg, 1995) and the functioning of multiple physiological systems such as stress reactivity and intersubjectivity (Feldman, Singer, & Zagoory, 2010; Hofer, 1994; Meaney, 2001; Trevarthen, 1998; Weaver, Szyf, & Meaney, 2002), endocrine systems (e.g., oxytocinergic system; Carter & Porges, 2013), and, in the long term, epigenetic controls of personality traits like anxiety (Champagne, 2014; Meaney, 2001). As moral behavior requires healthy self-regulation combined with an ability to focus on the needs of others in addition to the self, we hypothesized that early touch experience would be associated with characteristics connected to moral development. We used the evolved developmental niche (EDN) as a framework for thinking about the kind of touch-related care that might support optimal moral development.

### Evolved developmental niche

Every animal has a niche, or nest, for its young that aligns with the genotypic maturational schedule of the offspring and is important for optimizing development (Gottlieb, 2002). For social mammals, who emerged over 30 million years ago with intensive parenting, the nest includes extensive affectionate touch, along with breastfeeding on request, and self-directed play (Konner, 2005). The human niche is particularly comprehensive as babies are born highly immature relative to other species, and so additional nest components include multiple responsive caregivers and positive support. Ethologically, these components are necessary for a species-typical outcome. The presence of nest components—including touch—contributes to optimal development, and ties between touch and wellbeing have been found in human infants. For example, positive, affectionate touch (and lack of negative touch) is defined as part of a sensitive, responsive parenting style (Hane & Philbrook, 2012). Maternal carrying both decreases crying (Hunziker & Barr, 1986) and increases maternal responsiveness (Anisfeld, Casper, Nozyce, & Cunningham, 1990). Ties have also been established between massage and extensive skin-to-skin contact and improvements in preterm infants’ growth, development, and well-being (Feldman, Eidelman, Sirota, & Weller, 2002; Field, 1985). We wondered whether touch might be particularly important for aspects of wellbeing that relate to social capacities, such as moral development.

### Touch attitudes and behavior and moral development

Social mammals evolved to prefer staying close to a mother or allomother in early life, and young human offspring typically receive extensive positive touch from one or more adults (Hrdy, 2009; Konner, 2005). Animal experiments show that early experience with physical affection influences neurobiology and social capacities later (e.g., Henry, Richard-Yris, Tordjman, & Hausberger, 2009; Kuhn & Schanberg, 1998). This is true for humans too (Martin, Spicer, Lewis, Bluck, & Cork, 1991; Trickett & McBride-Chang, 1995). For example, affectionate touch attenuates infants’ stress responses (Feldman et al., 2010). Presence of sensitive and responsive touch also fosters secure attachment, which is related to later social functioning (for a review, see Cushing & Kramer, 2005). Consequently, early touch may play a role in establishing sociality—the capacity to enjoy flexible reciprocal relations with others—which leads to a prosocial, moral orientation toward others. In contrast, low positive touch or high negative touch may undermine the development of neurobiological systems that support sociality, leading instead to stressful social relations represented by withdrawal or aggression. These experiences may become hardened into “moral temperaments.” If indeed touch leads to a prosocial orientation toward others, we would expect to find positive correlations between positive touch attitudes and behaviors on the part of parents and sociomoral outcomes in the child. However, the substantial overlap between positive touch and a generally sensitive, responsive parenting style suggests that understanding the effects of touch would require partialling out the effects of responsive parenting. Consequently, wherever possible, we controlled responsivity in appraising correlations between parental touch and child sociomoral outcomes.

Examination of the relation between early touch and moral development requires separate examination of positive, affectionate touch and of negative touch (e.g., corporal punishment). Like absence of positive touch, presence of negative touch, such as corporal punishment, is associated with negative outcomes. Spanking young children is linked to later aggression, even after controlling for confounding factors such as abuse, domestic violence, stress, depression, and substance use (Taylor, Manganello, Lee, & Rice, 2010), and leads to increases in aggression over time, even when controlling for child trait aggression (for a review, see Gershoff, 2013). Spanking is associated linearly with a lifetime prevalence of psychiatric disorders, including anxiety, alcohol abuse, and externalizing disorders (Gershoff, 2002; MacMillan et al., 1999). Longitudinal studies with different ethnicities show that spanking predicts an increase in aggression no matter the background of the child (Berlin et al., 2009; Gershoff, Lansford, Sexton, Davis-Kean, & Sameroff, 2012). If touch plays a significant role in social processes such as responding to perceived threats, developing secure relationships, and manifestations of aggression, then early life positive and negative touch experiences would hypothetically be relevant for the development of interpersonal processes such as morality.

## Present studies

We conducted several studies. First, we examined maternal touch attitudes and behaviors in relation to child wellbeing generally and aspects of moral development (e.g., empathy, moral orientation) in particular. We conducted a second study examining longitudinal evidence for the effects of touch on moral development in childhood, and a final study examining retrospective reports of touch in childhood in relation to adult outcomes. We explored whether different patterns of association between child outcomes and parental attitudes versus behaviors pertaining to physical affection (positive touch) and corporal punishment (negative touch) would emerge and whether there were different patterns for positive and negative touch. We started with a study looking at parental touch attitudes in relation to children’s contemporaneous sociomoral behavior. Maternal attitudes are another form of communication that are typically correlated with behavior (Holden & Buck, 2002). We were interested to know whether attitudes alone, as an indirect form of communication, could predict child outcomes.

## Study 1

We investigated whether maternal touch attitudes are related to children’s wellbeing and sociomoral outcomes. As noted above, most research focuses on the role of touch behavior in avoiding or mitigating negative outcomes, but we wanted to investigate whether maternal attitudes alone would show similar associations with outcomes. We also examined sociomoral orientation.

Human behavior is influenced by individuals’ expectations about social interactions and interpretations of the social world. These expectations and interpretations may be prosocial, antisocial, or even some combination, so we looked at a full range of possibilities using the framework provided by triune ethics meta-theory (TEM; Narvaez, 2008, 2014, 2016). TEM suggests that morality is shaped initially by epigenetic and plasticity effects of early life care on neurobiological structures that underpin moral functioning (e.g., vagal tone; Porges, 2011). Theoretically, those who develop with sub-optimal care will be more likely to be stress reactive and move into self-protective moral orientations in social situations, whereas those whose care is closer to optimal will be well-regulated and likely to engage prosocially with others. The TEM framework for understanding moral development and behavior focuses on three ethics: Protectionism, Engagement, and Imagination. A Protectionist orientation focuses on self-preservation through social dominance or withdrawal. An Engagement orientation refers to prosocial attunement between self and others, and an Imagination orientation adds intention and creativity into social relations, allowing for an imaginative perspective beyond face-to-face interaction.

Theoretically, the sociomoral orientations of TEM relate to touch through neurobiological mechanisms. For example, lack of touch can increase stress, which is related to a self-focused orientation in social relations (Sapolsky, 1994) such as in Protectionism. In contrast, nurturing touch facilitates an oxytocin release linked to mellow mood and calm interaction (Fredrickson & Losada, 2005), facilitating prosocial relations as in Engagement and creative social relations as in Imagination. We thus hypothesized that mothers’ affirming attitudes toward positive touch and attitudes rejecting corporal punishment would be positively related to children’s development of an Engagement and/or Imagination orientation as well as other prosocial behaviors (e.g., empathy), but negatively related to Protectionism, misbehavior, or psychopathology.

### Method

#### Participants

Participants included 156 mothers (*M* = 33.82 years, *SD* = 5.10, range 18 to 48) of 3- to 5-year-olds (59% boys) recruited regionally from the USA. The majority of participants were married (92.3%), Euro-American (82.1%), and educated (21.8% with an associate’s degree or less schooling, 41.0% with Bachelor’s degree, and 37.2% with post college training). Yearly household income varied; 3.8% earned less than $15K per year, 13.5%: $15K–30K, 17.3%: $30–50K, 22.4%: $50–75K, 17.9%: $75–100K, 25.0%: over $100K.

#### Design and procedures

In this cross-sectional study, measures were administered through a single-session online questionnaire using Qualtrics. Mothers reported on their own touch attitudes and behavior as well as their child’s socio-moral behavior, moral orientation, and psychopathology. The survey link was disseminated through flyers at preschools, parenting listservs, and e-notices sent out by parenting organizations and a parenting blog. Participants were compensated with a $10 gift card to Amazon.

#### Measures

##### Touch attitudes

The scale included five statements about positive touch (e.g., “Holding or hugging target child when he or she is distressed,” α = .83) and three about corporal punishment (e.g., “Spanking target child with a belt or another instrument when needed,” α = .93) measured on 5-point Likert-type scales (1 = never how I parent, 5 = always how I parent). Each item was asked in reference to touch attitudes when the child was a baby and again in reference to current attitudes; responses were averaged.

##### Responsivity

Maternal responsivity was measured using five items with a 5-point scale (1 = Strongly Disagree, 5 = Strong Agree; α = .87) that assessed attitudes about the importance and wisdom of responding sensitively to infants’ needs (e.g., “Parents who respond quickly to a baby spoil the baby;” reverse scored).

##### Child outcome measures

Child outcomes were measured via maternal report using a combination of new and standardized measures.

##### Sociomoral behavior

To measure sociomoral behavior, we used the *empathy* and *concern* subscales of *My Child* (Kochanska, DeVet, Goldman, Murray, & Putnam, 1994; *α*s = .86 and .88 for this study respectively) and the Children’s Behavior Questionnaire inhibitory control subscale (*α* = .83; Rothbart, Ahadi, Hershey, & Fisher, 2001). The rest of the measures were from Gleason, Narvaez, Cheng, Wang, and Brooks (2016). These included frequency of *misbehavior* (six items; e.g., “How often does your child misbehave?” *α* = .75) using a 5-point Likert-type scale (never, once a week or less, several times a week, every day, several times a day); demonstrations of *happiness* (five items; e.g., “How often does your child…squeal with happiness,” *α* = .72) measured on a 6-point scale (1 = never to 6 = more than once a day); and *thriving* (14 items; e.g., “My child deals well with problems,” α = .91), scored using a 6-point scale (1 = never to 6 = always).

##### Moral orientation

We used the Child Triune Ethics Measure (CTEM; Gleason et al., 2016), an adaptation of the Triune Ethics Orientation measure for adults (Narvaez, Brooks & Mattan, 2011; Narvaez & Hardy, 2016; Narvaez, Wang & Cheng, 2016), to measure children’s sociomoral orientation. Parents rated how often they saw child behaviors in social situations using a 6-point Likert scale (1 = never to 6 = several times a day). Three of the seven subscales come from the ethic of Protectionism: (a) *Opposition* (ten items; α = .91; e.g., “oppositional”), (b) *Distrust* (four items; α = .58; e.g., “untrusting”), and (c) *Withdrawal* (ten items; α = .90; e.g., “timid”); three from Engagement: (d) *Social enjoyment* (nine items; α = .93; e.g., “affectionate”), (e) *Social attunement* (eight items; α = .89; e.g., “gentle”), (f) *Social consideration* (six items; α = .84; e.g., “respectful”); and one from Imagination: (g) *Social imagination* (six items; α = .83; e.g., “enterprising”).

##### Psychopathology

We used a 17-item *depression* frequency measure (Gleason et al., 2016; α = .92; e.g., “How often does your child lack confidence?”), employing a 6-point Likert-type scale (1 = never to 6 = several times a day). *Anxiety* was measured using the 27-item Preschool Anxiety Scale (Spence, Rapee, McDonald, & Ingram, 2001; e.g., “Is afraid of meeting or talking to unfamiliar people,” α = .94) rated on a 5-point Likert-type scale (0 = not true at all to 4 = very often true).

### Results

See Table 1 for means and standard deviations for all subscales.

**Table 1:** Study 1 scales, means, and standard deviations (*N* = 156)

	Scale	*M*	*SD*
Maternal touch attitudes			
Positive touch	1–5	4.66	.51
Anti-punishment	1–5	4.58	.73
Child sociomoral outcomes			
Prosocial			
Social enjoyment	1–6	5.59	.60
Social attunement	1–6	5.07	.74
Social consideration	1–6	5.04	.70
Social imagination	1–6	4.87	.83
Empathy	1–7	5.29	.91
Concern	1–7	4.85	1.16
Inhibitory control	1–7	4.97	.87
Thriving	1–5	4.38	.48
Happiness	1–6	5.14	.59
Antisocial			
Social opposition	1–6	2.89	1.01
Social distrust	1–6	3.04	.88
Social withdrawal	1–6	2.57	.84
Misbehavior	1–5	2.33	.63
Psychopathology			
Depression	1–6	2.58	.80
Anxiety	1–5	1.64	.58

**Fig. 1:**
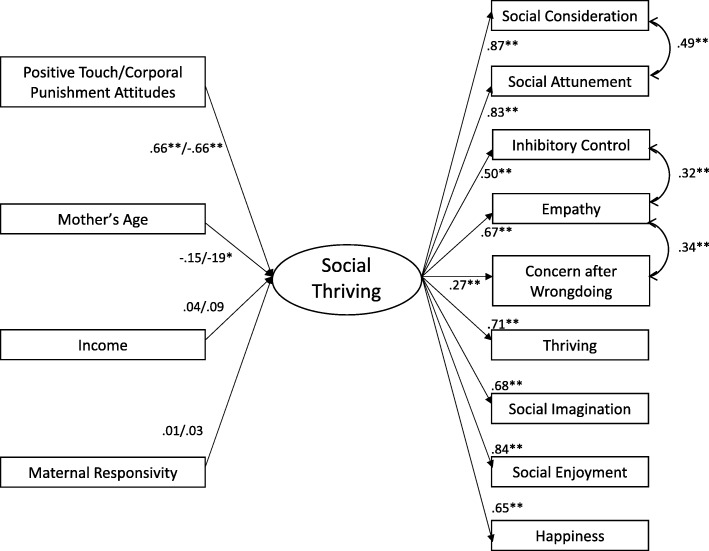
Social thriving predicted by touch attitudes, separate models. All coefficients are standardized. Coefficients for positive touch and corporal punishment attitudes are separated by “/” for all predictors. Latent variable loadings and covariances are constrained in both models and are the same for both models. Positive touch attitudes predicting social thriving fit indices: robust χ^2^(67) = 94.599, *p* = .015; robust CFI = .963; RMSEA = .051, 90% CI = [.025, .073]; SRMR = .054. Corporal punishment attitudes predicting social thriving fit indices: robust χ^2^ (67) = 145.120, *p* < .001; robust CFI = .902; RMSEA = .086, 90% CI = [.068, .105]; SRMR = .063

**Fig. 2:**
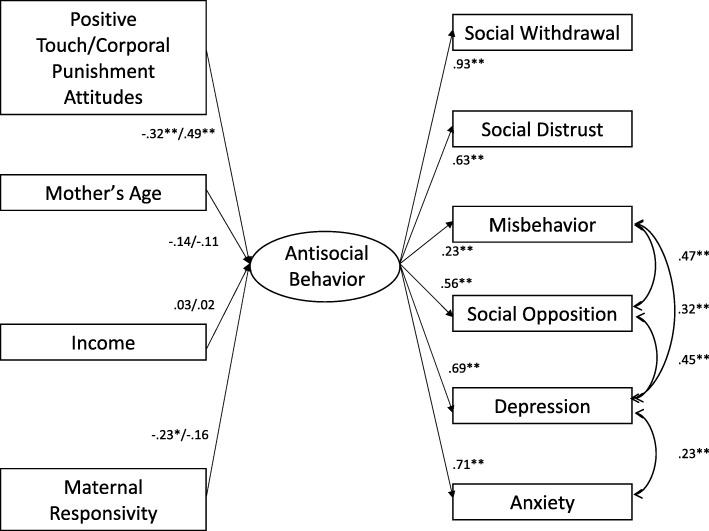
Antisocial behavior predicted by touch attitudes, separate models. All coefficients are standardized. Coefficients for positive touch and corporal punishment attitudes are separated by “/” for all predictors. Latent variable loadings and covariances are constrained in both models and are the same for both models. Positive touch attitudes predicting antisocial behavior fit indices: robust χ^2^ (34) = 56.791, *p* = .008; robust CFI = .942; RMSEA = .066, 90% CI = [.035, .093]; SRMR = .055. Corporal punishment attitudes predicting antisocial behavior fit indices: robust χ^2^ (34) = 65.569, *p* = .001; robust CFI = .921; RMSEA = .077, 90% CI = [.051, .103]; SRMR = .058

Based on prior research (Gleason et al., 2016; Narvaez et al., 2016; Narvaez et al., 2019), we conceptualized moral orientations and outcomes as having two factors, one representing positive outcomes and the other representing negative outcomes: *Social Thriving* (social consideration, social attunement, inhibitory control, empathy, concern after wrongdoing, thriving, social imagination, social enjoyment, and happiness); and *Antisocial Behavior* (social withdrawal, social distrust, misbehavior, social opposition, depression, and anxiety). Then we fit two separate models to the data, one for each latent construct, using confirmatory factor analysis. The measurement models of outcomes had good fit (Robust χ^2^s: 34.341 [*df* = 24, *p* = .079], 6.472 [*df* = 5, *p* = .263]; CFIs: .983, .994; RMSEAs: .054, .044; SRMRs: .043, .024, respectively). We used these measurement models as latent outcomes predicted by positive touch attitudes and corporal punishment attitudes, controlling for maternal responsivity, age, and income. The factor mean for each latent variable was fixed to 0. The factor standard deviation for the first factor was 3.638, and the factor standard deviation for the second factor was 7.825. See Figs. 1 and 2 for fit indices and factor loadings for these models.

In general, positive touch and corporal punishment attitudes predicted the two latent outcomes. Positive touch attitudes positively predicted social thriving (*B* = .66, *p* < .01), whereas corporal punishment attitudes negatively predicted social thriving (*B* = − .66, *p* < .01). Further, positive touch attitudes negatively predicted antisocial behavior (*B* = − .32, *p* < .01) and corporal punishment attitudes positively predicted antisocial behavior (*B* = .49, *p* < .01). In all models, when compared to control variables, the touch attitude variables had the greatest magnitude of coefficients that predicted the latent variable outcomes (see Figs. 1 and 2).

### Discussion

Our hypothesis, that maternal touch attitudes would influence both children’s prosociality and wellbeing, and antisocial behavior and psychopathology, was supported. Mothers’ affirming attitudes toward positive touch coincided with the development of a social orientation that includes engagement with others, empathy, moral imagination, and low rates of misbehavior and psychopathology. The endorsement of corporal punishment was similarly related to child outcomes; that is, stronger punishment attitudes related to lack of engagement, imagination, and higher rates of internalizing and externalizing psychopathology.

These models—particularly their strength even when controlling for age, income, and responsivity—emphasize how a mother’s attitude toward touch relates to her reports of her child’s behavior. Parenting attitudes and behavior are not always perfectly aligned (Holden & Buck, 2002), but according to these results, mothers’ abstract intentions with regard to physical affection are associated with their perceptions of their children’s view of the social world and behavior within it. The findings suggest that maternal attitudes communicate in ways important to child wellbeing and moral development. However, the homogeneous sample means these results have limited generalizability. In addition, although we included reports of attitudes toward touch in infancy and currently, these measures were combined rather than studied as they developed over time. Consequently, we examined effects of maternal touch behavior longitudinally in study 2.

## Study 2

Our second study expanded our understanding of the relationships among touch and child sociomoral outcomes and well-being in three ways. First, we used longitudinal data from 4–36 months to examine effects of early touch on later outcomes. Second, in addition to self-reports of parenting behaviors, observations of mother-child interaction and interviews were conducted at each time point. Lastly, data were gathered from a racially and socio-economically diverse sample. In this context, we examined the influence of maternal touch attitudes and behavior on child prosociality and behavior problems, hypothesizing that the relations found in the first study would be replicated and that we would find longitudinal effects of early life touch on later sociomoral outcomes.

### Method

#### Design and procedures

Data were obtained from the Parenting for the First Time project (PFT; Borkowski et al., 2012; Lefever et al., 2008), a 3-year longitudinal (prenatal to age 3) study of mother-child dyads at risk for child neglect. First-time mothers and their children were recruited in their last trimester of pregnancy from primary care facilities during 2002–2005 in four US cities: Birmingham, AL; South Bend, IN; Washington, DC; and Kansas City, KS/MO. The subset of variables from the PFT data set used included data from interviews conducted when infants were 4, 6, 8, 18, 24, 30, and 36 months old. The interviews at 4, 8, 18, and 30 months were generally conducted in the home and included observations of mother-child interaction and the home environment. At 24 and 36 months, the mother was interviewed concerning her child’s socio-emotional development.

#### Participants

The total sample (*N* = 682) had 396 adolescent mothers (*M*_age_ = 17.5 years, *SD* = 1.12 years), 169 adults with limited education beyond a high school diploma (*M*_age_ = 25.5 years, *SD* = 3.0) and a comparison group of 117 adults with at least 2 years of college (*M*_age_ = 27.9 years, *SD* = 3.9). Ethnicities were 65% African-American, 19% European-American, 15% Hispanic-American, and 1% other. At the prenatal interview, 61% were single, 16% married, and 22% living with a partner; 22% were employed at the prenatal visit and 49% worked until pregnancy. Across dyads, 49.9% of children were male. Although the retention rate at 36 months was low (55%), no significant differences emerged in demographics (group, mother’s race, age, or education, child’s gender, birth weight/length) between mothers who did and did not complete at least a portion of the 36-month assessment.

#### Measures

##### Family demographics

Information about maternal age, race, education level, and household income-to-needs ratio (household income divided by number of people supported) were gathered via maternal interview and used as covariates (Lamb & Ahnert, 1998; National Institute for Child Health and Human Development Early Child Care Research Network, 2001). In addition, we used two dummy coded variables to code the three groups: teen vs. adult high-education mothers, and adult low-education mothers vs. adult high-education mothers.

##### Touch attitudes and behaviors

To measure *attitudes towards punishment*, we used eight items from a punishment subscale of the Parenting Style questionnaire (O'Callaghan, Borkowski, Whitman, Maxwell, & Keogh, 1999; Sommer et al., 1993; Sommer et al., 2000) collected via maternal interview when the child was 6 months old. This measure captured the mother’s knowledge and endorsement of a variety of parenting practices and was adapted from the Adult-Adolescent Parenting Inventory (Bavolek, 1984). Mothers rated items on a 5-point scale representing the degree to which she endorsed the behavior (α = .86; e.g., “Children should always be spanked when they misbehave”); lower scores represented endorsement of punitive parenting behaviors. We measured maternal touch behavior using items (each coded as 0 vs. 1, higher scores being more positive) selected from the Infant/Toddler (4, 8, and 18 months) and the Early Childhood (30 months) versions of the *Home Observation for the Measurement of the Environment* (HOME; Caldwell & Bradley, 1984, 2001). HOME is a checklist (45 items) completed during observations and parent interview during a lengthy in-home visit. Inter-rater reliability was established using video and subsequent live interviews until a criterion of 90% agreement was reached and fidelity was reassessed every 6 months throughout the course of the project. *Lack of punishment behavior* was collected at 4 months (α = .31), 8 months (α = .35), 18 months (α = .39), and 30 months (α = .39); items were (1) no more than one instance of corporal punishment, (2) does not slap or spank the child, and (3) does not interfere or restrict the child more than twice. *Positive touch behavior* was also collected at 4 months (α = .12), 8 months (α = .20), 18 months (α = .32), and 30 months (α = .37); items were (1) parent picks up child regularly when not sleeping, and (2) parent caresses or kisses child at least once during visit.

##### Maternal responsivity

We used the responsivity subscale from the HOME (11 items; α = .69), which reflects the mother’s verbal and affective responsiveness to the child and verbal responses during the interview.

##### Child outcomes

Direct assessment of mother-child interaction came from 20-min naturalistic observations of mother-child pairs during the 18- and 30-month home visits. Mothers were instructed to do what they would normally do with the child and to pretend that the interviewer was not there. The dyad was observed for four 5-min observation intervals with time between intervals for rating both mother and child along several dimensions based on a coding schema developed by Landry, Smith, Miller-Loncar, and Swank (1997). Our analyses focused on three child variables: behavioral regulation, social engagement, and cooperation. Each dimension of child behavior was rated on a 5-point scale; higher scores indicated more positive behavior. Interviewers were trained to 80% reliability with a master coder during videotaped and in vivo observations (Hammond, Landry, Swank, & Smith, 1999); fidelity was reevaluated every 6 months.

The Infant-Toddler Social and Emotional Assessment (ITSEA; Carter & Briggs-Gowan, 2006; Carter, Briggs-Gowan, Jones, & Little, 2003) was used to measure children’s externalizing (α = .87) and internalizing (α = .81) behavior problems and competence (α = .87) at 24 and 36 months. Mothers responded to 102 items using a 3-point scale (*not true*/*rarely* to *very true*/*often*)*.*

## Results

Among the maternal variables, ratings of positive touch decreased significantly from 4 to 30 months, *t*(291) = 12.11, *p* < .001, Cohen’s *d* = .71, and punishment increased significantly from 4 to 30 months, *t*(291) = 9.05, *p* < .001, Cohen’s *d* = .53. Means for child outcomes varied little between time points; to the extent that changes are identifiable they indicate more prosocial and less antisocial behavior with age. In general, observations of children’s behavior were on the upper end of the range for prosocial behaviors and the lower end for behavior problems (see Table 2 for all means and standard deviations).

**Table 2:** Study 2 descriptive statistics for punishment attitudes, touch behaviors, and child outcomes

Variable	*N*	Range	*M*	*SD*
Maternal variables				
6-month anti-punishment attitudes	425	8–40	31.05	5.96
4-month positive touch behavior	466	0–2	1.82	.41
8-month positive touch behavior	406	0–2	1.66	.55
18-month positive touch behavior	391	0–2	1.43	.68
30-month positive touch behavior	359	0–2	1.27	.75
4-month lack of punishment behavior	466	0–3	2.77	.50
8-month lack of punishment behavior	406	0–3	2.53	.70
18-month lack of punishment behavior	390	0–3	2.19	.87
30-month lack of punishment behavior	359	0–3	2.25	.85
Child outcomes				
18-month behavioral regulation	369	1–5	4.48	.65
30-month behavioral regulation	342	1–5	4.62	.62
18-month social engagement	368	1–5	3.62	1.02
30-month social engagement	341	1–5	3.63	1.23
18-month cooperation	363	1–5	4.11	.84
30-month cooperation	341	1–5	4.29	.86
24-month externalizing	415	0–2	.70	.32
36-month externalizing	363	0–2	.61	.31
24-month internalizing	412	0–2	.63	.23
36-month internalizing	360	0–2	.58	.25
24-month competence	406	0–2	1.32	.27
36-month competence	357	0–2	1.39	.30

For punishment behavior, higher scores = less corporal punishment

We hypothesized that maternal touch attitudes and behavior would predict both concurrent as well as future sociomoral outcomes. We ran partial correlations among punishment attitudes (at 6 months) and touch behaviors (at 18 and 30 months only, because the variations in touch behaviors in 4 and 8 months were small) and child outcomes while controlling for responsivity, maternal age and education, and income-to-needs ratio (see Table 3). Mothers whose attitudes rejected negative touch at 6 months subsequently had toddlers who were more socially engaged at 18 months; these same children were more competent and less likely to have behavioral problems at 24 months. However, these effects disappeared when measured a year later. Positive touch parenting behaviors at 18 months positively correlated with concurrent but not future behavioral regulation and with social competence at both 24 and 36 months. Positive touch parenting behaviors at 30 months positively correlated with concurrent social engagement and lower externalizing problems 6 months later. Lack of negative touch was positively related to concurrent behavioral regulation at both 18 and 30 months. Mothers’ lack of negative touch behaviors at 18 months were also positively related to 36-month ratings of social competence, and negatively to children’s externalizing problems at 24 and 36 months. However, by 30 months, maternal avoidance of punishing touch was significantly related to all of the child outcomes except internalizing problems.^1^ The statistically significant correlations were in the small to medium size based on Cohen’s rule of thumb (small, .10; medium: .30; Cohen, 1992).

**Table 3:** Study 2 partial correlations among maternal anti-punishment attitudes, touch behavior, and child sociomoral outcomes

Child outcomes by age	Anti-punishment attitudes	Positive touch		Lack of negative touch	
	6 months	18-months	30-months	18-months	30-months
18-month behavioral regulation	.02 (.75)	*.15* (.*03*)	.07 (.33)	.*24* (< .*001*)	.*19* (.*01*)
18-month social engagement	.*23* (.*001*)	.10 (.14)	.09 (.22)	.10 (.15)	.*20* (.*004*)
18-month cooperation	.02 (.82)	-.03 (.71)	.04 (.61)	.08 (.27)	.*17* (.*02*)
30-month behavioral regulation	.08 (.27)	.11 (.13)	.07 (.31)	.02 (.77)	.*30* (< .*001*)
30-month social engagement	.13 (.07)	.06 (.44)	.*24* (.*001*)	.04 (.57)	.*22* (.*002*)
30-month cooperation	.04 (.62)	− .01 (.86)	.09 (.23)	− .03 (.69)	.*28* (< .*001*)
24-month externalizing	− .*20* (.*002*)	− .04 (.56)	.01 (.92)	− .*14* (.*04*)	− .*19* (.*01*)
24-month internalizing	− .*15* (.*03*)	.03 (.61)	.03 (.72)	− .02 (.80)	− .07 (.33)
24-month competence	.*18* (.*01*)	.*14* (.*04*)	.11 (.14)	.09 (.20)	.*17* (.*02*)
36-month externalizing	− .11 (.12)	− .03 (.67)	− .*16* (.*02*)	− .*14* (.*04*)	− .*14* (.*04*)
36-month internalizing	− .11 (.13)	.04 (.57)	− .09 (.21)	− .12 (.08)	− .07 (.36)
36-month competence	.13 (.07)	.*22* (.*002*)	.11 (.13)	.*15* (.*04*)	.*15* (.*04*)

Correlations control for responsivity, income-to-needs ratio, maternal age, and education. High scores on anti-punishment attitudes indicate rejection of corporal punishment

Significant values are set in italics. *P*-values are in parentheses

### Discussion

The partial correlations that emerged between mothers’ positive touch behaviors at 18 and 30 months and child outcomes suggest two conclusions. First, the influence of positive touch on behavioral regulation might wane over time, as regulation at 30 months was not related to current or earlier positive touch. As the child grows older, touch may become relatively less important because children understand the meaning of the words mothers use to express affection and support. Second, given the differential pattern of correlations between positive touch at 18 and 30 months and child outcomes, the effects of positive touch might change over time. Earlier positive touch connects to social competence, but the effects of touch in the third year relate to social engagement and fewer externalizing behavior problems. Taken together, these findings suggest that positive touch initially affects self-regulation, and continuing positive touch in turn aids children’s developing social competence. The positive touch associated with higher competence and engagement at ages 2 to 2.5 might help attenuate outward demonstrations of misbehavior at age 3, perhaps because of the power of oxytocin, which rises with parental touch (Feldman, 2007), in promoting calming. Generally, however, relations between positive touch and child outcomes were few.

The idea that touch might first operate on social outcomes through regulation is supported by the findings related to punishing (negative) touch. Mothers who engaged in corporal punishment with their toddlers had children who were less regulated, less socially engaged, less cooperative, less socially competent, and had more externalizing problems. One interpretation of these findings is that such negative touch communicates rejection to the child, the “badness” described by Prescott (1996).

The findings from this study reinforce the notion that touch attitudes and behaviors relate in subtly different ways to child outcomes, and that affectionate and punishing touch (or lack thereof) are also different sources of influence on children’s behavior. For example, attitudes related to 18-month social engagement and primarily to social behavior at age 2, but not to measures of behavioral regulation. Touch behaviors, on the other hand, related to regulation, and for negative touch, to some measures of social behavior as well. Perhaps mothers’ early attitudes influence a child’s orientation toward others and some social behavior, but the influence of parenting touch behavior on regulation has greater implications for child outcomes. The results also raise the question of the influence of change over time in both attitudes and behavior. The means for lack of negative touch dropped between 8 and 18 months (meaning engagement in negative touch increased), but we did not have data on whether parent touch attitudes changed as well over that time period.

Changes in parental behavior over time could reflect changes in attitudes and/or reflect the developing capacities of the child. As the child becomes an ever-greater participant in the parent-child relationship, those parents whose children were not well-regulated in infancy and toddlerhood might have been more willing to turn to physical forms of punishment. This idea is supported by the positive correlations between the 18-month child outcomes and lack of negative touch shown by parents a full year later. As for the contributions of positive versus negative touch, on the one hand, both variables predicted children’s behavioral regulation, echoing the findings in the literature connecting touch practices generally to the physiological systems that underlie behavior, such as vagal tone (Eisenberg & Eggum, 2008; Porges, 2011). On the other hand, overall, a *lack* of negative touch appeared to be a better predictor than provision of positive touch for both prosocial and problematic child behaviors. Moreover, the few connections that did emerge between positive touch and prosocial behaviors, such as social competence and fewer behavior problems, did so only after a 6-month lag—positive touch at 18 and 30 months were unrelated to concurrent measures of children’s social engagement and cooperation. This pattern also held true for a lack of negative touch at 18 months, but by 30 months, lack of negative touch was positively associated with concurrent prosocial child outcomes as well as lower externalizing behaviors and higher competence at 36 months. These findings suggest that early patterns of caregiver touch behavior relate to the development of children’s self-regulation and whether parents begin to use punishing touch.

## Study 3

The first two studies examined how maternal touch attitudes and behavior communicate support for or undermine children’s moral development. We established relations between touch and child outcomes, but theoretically touch might have implications for adult functioning as well. If indeed the positive and negative touch attitudes and behaviors of caregivers in early childhood establish physiological systems and psychological patterns that are carried forward in development, then relationships ought to emerge between adult reports of early childhood touch experiences and adult contemporaneous functioning. Certainly, adversity in childhood has lasting effects into adulthood (Anda et al., 2006), but an equally interesting question is whether smaller variations in experience from a nonclinical sample would show similar relations between early experience and adult moral psychology. In particular, we wondered whether reports of early life touch could be connected to adult moral orientations as defined by Triune Ethics Meta-Theory. In addition to examining relations to adult functioning, we also wanted to explore mechanisms influencing *how* the relationship between touch and moral orientation might be established.

In thinking about how touch might relate to moral orientation, we reasoned that the pathway might be indirect. Specifically, we expected that early touch experiences, because of their apparent relation to fundamental physiological systems, might influence psychological wellbeing. As attachment security and mental health are well-established elements of psychological well-being (Mikulincer & Shaver, 2007) and correlates of moral development (Narvaez et al., 2016), we chose these variables as possible mediators of the relation between early life touch and moral orientation. We also wondered whether specific capacities, such as empathy and perspective-taking, which are important for moral development and influenced by psychological wellbeing (Shanafelt et al., 2005), might play a role in the relation between touch and moral orientation as well. Specifically, we hypothesized that the effects of attachment security and mental health on moral orientation might well be mediated by these moral capacities. After all, attachment security has been associated with an individual’s capacity to take another’s perspective (Mikulincer & Shaver, 2007) or to feel empathy (Wei, Liao, Ku, & Shaffer, 2011). Consequently, we created a sequential mediational model that allowed us to test for relations between retrospective reports of caregiver touch in early childhood and adult moral orientations via psychological wellbeing (e.g., attachment, mental health) and moral capacities (e.g., empathy). We expected that since links between adjacent nodes in this model have been shown to be significant in prior research, we might find overall sequential mediation effects.

### Method

#### Participants

The sample consisted of 607 adults (*M*_age_ = 28.39 years, *SD* = 11.23; 51.4% male). The racial/ethnic composition of the sample was 69% Euro-American, 15% Asian American, 6% African American, 6% Hispanic/Latino, and 4% Native American.

#### Design and procedures

In this retrospective study, participants were recruited from introductory psychology courses at a private USA Midwestern university and from Amazon Mechanical Turk. Participants completed on-line surveys pertaining to their early childhood experiences as well as current attachment, mental health, moral orientations, and moral capacities. Students received course credit and Amazon Turk participants were paid about $5.00/h for their completion of the survey, which took on average fewer than 30 min.

#### Measures

##### Childhood touch

Positive and negative touch from caregivers were evaluated using one question each on the evolved developmental niche history (EDNH; Narvaez, Wang, & Cheng, 2016), a measure that assesses adult ratings of childhood experience (before age 18) consistent with evolved human mammalian caregiving (Konner, 2005). The questions (using a 5-point Likert-type scale: 1 = Never, 5 = Very often) were: How often were you affectionately touched, kissed, or hugged by at least one of your parents or guardians? (positive touch) and Did you ever receive corporal punishment from a parent or guardian (e.g., hit, spanked, slapped, pinched)? (reversed; lack of corporal punishment).

##### Attachment

The secure attachment item from the Close Relationship Questionnaire (CRQ; Bartholomew & Horowitz, 1991) was used as a proxy for measuring secure attachment. Participants rated (1 = not at all like me, 7 = very much like me) the description of secure attachment: “It is relatively easy for me to become emotionally close to others. I am comfortable depending on others and having others depend on me. I don’t worry about being alone or having others not accept me.”

##### Mental health

We measured mental health, specifically, internalizing psychopathology, with the 64-item Inventory of Depression and Anxiety Symptoms (IDAS; Watson et al., 2007; *α* = .97) excluding suicidality because data collection occurred online anonymously, without the possibility for implementing a safety protocol. Participants indicated the degree to which they have experienced symptoms over the past 2 weeks using a 5-point Likert scale (1 = *not at all* to 5 = *extremely*). Higher scores indicate poorer mental health.

##### Moral capacities

To measure moral capacities, we used three subscales from Davis’s (1983) Interpersonal Reactivity Index, empathic concern (six items, *α* = .84), perspective-taking (seven items, *α* =.87), and personal distress (seven items, *α* = .85), all answered with a Likert-type scale (1 = not at all like me, 7 = just like me). Empathic concern measures the tendency to experience warm and compassionate feelings for others; perspective-taking measures the tendency to consider other people’s point of view; and personal distress reflects a tendency to feel excessive discomfort when others are in pain (focusing concern on the self instead of the victim).

##### Moral orientations

Three moral orientations, engagement, social opposition, and social withdrawal, were assessed using the adult version of the Triune Ethics Orientation measure described in study 1 (Narvaez & Hardy, 2016). For each orientation, a set of four characteristics was presented (Engagement: caring, compassionate, merciful, cooperative; Social Opposition: combative, vigilant, belligerent, fierce; Social Withdrawal: submissive, yielding, timid, unassertive), and participants were asked to rate four statements with respect to the set (e.g., “I strongly desire to have these characteristics;” “my family thinks I have these characteristics”) on a 5-point Likert-type scale (1 = *strongly disagree*, 5 = *strongly agree*). Responses across statements were averaged, and Cronbach’s alphas were high (engagement: *α* = .89; social opposition: *α* = .92; social withdrawal: *α* = .87).

### Results

**Fig. 3:**
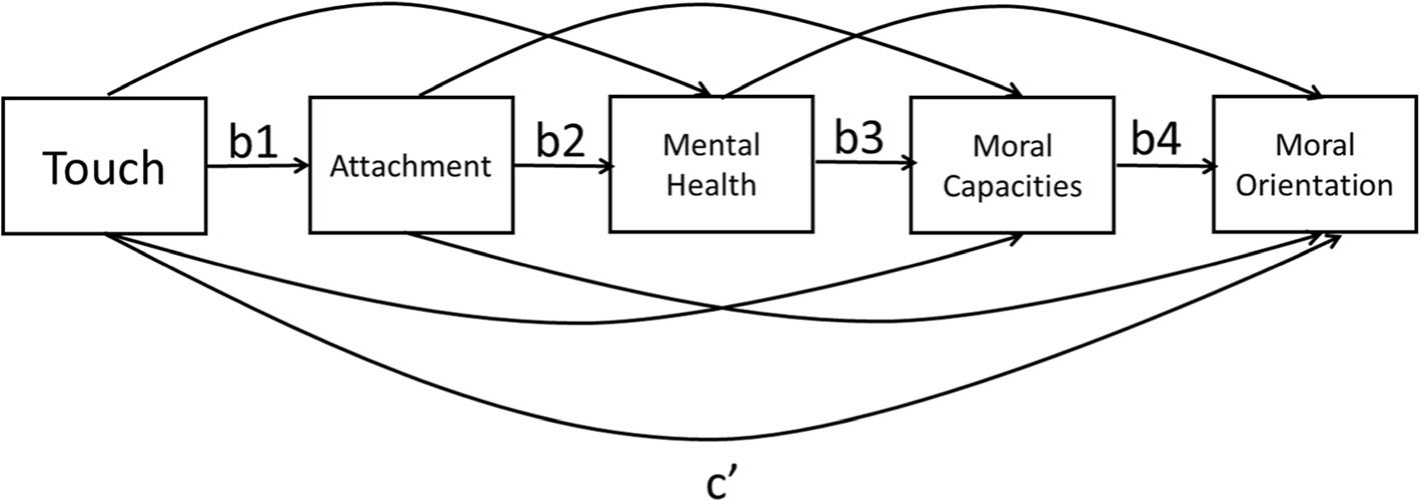
Theoretical model for mediation across constructs: from touch (positive touch and lack of corporal punishment in childhood) to attachment (security), mental health (anxiety and depression), and moral capacities (empathy, perspective-taking, or personal distress) and then to moral orientation (social engagement, or self-protectionism as social withdrawal or social opposition)

Descriptive statistics for all variables are reported in Table 4. We wanted to assess whether early experiences with touch would relate to adult wellbeing and morality. We proposed a mediation model connecting touch (positive touch or lack of corporal punishment) to moral orientation through security of attachment, mental health, and moral capacities (empathy, perspective-taking, and personal distress; see Fig. 3). We tested several versions of this model.^2^

**Table 4:** Study 3 Means and standard deviations for touch reports and adulthood measures

	Range	*M*	SD
Positive touch	1–5	3.71	1.04
Lack of corporal punishment	1–5	3.63	1.03
Secure attachment	1–7	4.47	2.07
Poor mental health (anxiety and depression)	1–5	3.51	1.33
Empathic concern	1–5	3.74	.74
Perspective-taking	1–5	3.57	.68
Personal distress	1–5	2.54	.79
Social engagement	1–4	4.13	.69
Social opposition	1–4	1.86	.92
Social withdrawal	1–4	2.11	.94

We hypothesized that the relation between touch and moral orientation would be mediated by wellbeing (i.e., attachment security and mental health) and moral capacities (i.e., empathy, perspective-taking, and personal distress). This hypothesis was partially supported for each of the moral orientations (see Table 5 for supported models). For engagement, all the path coefficients between adjacent variables were significant and the directions were consistent with our expectations for both empathy and perspective-taking, but not for personal distress. More specifically, we found significant positive path coefficients between reports of both positive touch and lack of corporal punishment in childhood with attachment in adulthood, negative path coefficients between attachment and poor mental health and between poor mental health and moral capacity (empathy or perspective-taking), and positive path coefficients between both moral capacities and engagement orientation. In addition, the overall indirect effects, a measure of the overall sequential mediation effect, were significant (based on the 95% bias corrected bootstrap confidence intervals), indicating that the sequence of mediators significantly explained the relation between touch and the moral orientation of engagement. Furthermore, both types of touch were still significantly related directly to engagement after considering the sequence of mediators, indicating partial mediation.

**Table 5:** Study 3 path coefficient estimates (and *p* values) among moral orientations and touch variables

	b1	b2	b3	b4	Indirect effect	c′
Social engagement	Moral capacity: empathy					
Positive touch	.46 (< .001)	− .15 (< .001)	− .04 (.03)	.52 (< .001)	.002 (.03)	.06 (.01)
Lack of corporal punishment	.28 (< .001)	− .16 (< .001)	− .04 (.02)	.51 (< .001)	.001 (.06)	.07 (.002)
	Moral capacity: perspective-taking					
Positive touch	.46 (< .001)	− .15 (< .001)	− .05 (.002)	.29 (< .001)	.001 (.01)	.08 (.001)
Lack of corporal punishment	.28 (< .001)	− .16 (< .001)	− .05 (.003)	.28 (< .001)	.001 (.03)	.11 (< .001)
Social opposition	Moral capacity: empathy					
Positive touch	.46 (< .001)	− .15 (< .001)	− .04 (.03)	− .36 (< .001)	− .001 (.03)	− .08 (.02)
Lack of corporal punishment	.28 (< .001)	− .16 (< .001)	− .04 (.02)	− .36 (< .001)	− .001 (.07)	.05 (.21)
	Moral capacity: perspective-taking					
Positive touch	.46 (< .001)	− .15 (< .001)	− .05 (.002)	− .24 (< .001)	− .001 (.03)	-.10 (.01)
Lack of corporal punishment	.28 (< .001)	− .16 (< .001)	− .05 (.003)	− .24 (<.001)	− .001 (.06)	− .07 (.06)
Social withdrawal	Moral capacity: personal distress					
Positive touch	.46 (< .001)	− .15 (< .001)	.18 (< .001)	.34 (< .001)	− .01 (.001)	− .07 (.03)
Lack of corporal punishment	.28 (< .001)	− .16 (< .001)	.18 (< .001)	.34 (< .001)	− .003 (.01)	− .07 (.13)

For the indirect effects, the *p* values are from Sobel tests. All 95% bias corrected bootstrap confidence intervals indicated that indirect effects included here were significant

Similar patterns emerged for social opposition with empathy or perspective-taking as moral capacity mediators, demonstrating negative relations. For both positive touch and lack of corporal punishment, the indirect effects were significant (based on the bootstrap method). Additionally, the direct connection between lack of corporal punishment and social opposition was not significant after the inclusion of the mediators, indicating complete mediation.

For social withdrawal, a significant mediation model also emerged through personal distress, but not empathy or perspective-taking. For both positive touch and lack of corporal punishment, poor mental health was positively related to personal distress. Personal distress was positively related to social withdrawal, and the significance of the overall indirect effects supported our hypothesized mediational process. The direct link between touch and social withdrawal after including the sequence of mediators was also significant for positive touch but not for lack of negative touch, indicating partial and complete mediation respectively.

### Discussion

Our interest was to examine whether reported touch experience in childhood related to adult aspects of wellbeing affected by touch (attachment, mental health) and whether these all influenced moral capacities and moral orientations. Our predictions were largely confirmed. In our models, positive early touch was predictive of adult wellbeing and moral capacities and moral orientations in expected directions.

The mediation models suggest three interesting avenues for further research. First, the success of these models suggests a pattern for linking early caregiving practices to psychological outcomes (attachment, mental health) that appear to be foundational for moral capacities and resulting moral orientations in adulthood. Research on the relation of touch to the development of attachment and mental health early in development could highlight the ways that each step in the development of moral orientations is constructed over time. Second, the fact that the same model fit both the engagement and social opposition moral orientations, albeit in opposite ways, implies a common etiology for these orientations, depending in particular on the development (or lack) of perspective-taking. The fact that failures of perspective-taking resulted in social opposition, whereas the social withdrawal orientation was connected to failures of emotion regulation (personal distress) that correlated positively with poor mental health, suggests that efforts to shift individuals away from the self-protective moral orientations toward engagement would need to be targeted to the protectionist orientation to which they were prone. Third, the cross-sectional data used in the study did not allow us to imply temporality and causality. Thus, future longitudinal studies are needed for testing the indirect effects temporally.

The findings, though preliminary, suggest that experiences with touch in early life may shape adult capacities for getting along with others and the type of ethical orientation they bring to social relationships—open or bracing. These outcomes may have profound implications for society in that children who lack affection or receive corporal punishment may grow into adults who are less capable of getting along with others cooperatively, and who withdraw or oppose others in social situations.

## General discussion

The goal of the research presented here was to test the hypothesis that touch in early childhood is related to sociomoral development and wellbeing later in life. Because of the evidence connecting caregiver touch to regulatory and social functions in humans and other social mammals (Field, 2010; Meaney, 2010), we postulated a role for touch in the development of psychosocial and moral functioning, an idea that was largely supported by studies presented in this paper. In addition, we explored whether positive, affectionate touch and negative, punishing touch have different patterns of relations with moral development; evidence for this idea was mixed across the studies presented here. Lastly, we explored whether caregivers’ attitudes and behaviors with respect to touch might also differentially relate to sociomoral outcomes. In the subset of studies that examined both attitudes and behaviors simultaneously, results suggest that this hypothesis might deserve further examination.

### Touch and moral development

In each of our three studies, we found evidence suggesting that early maternal touch orientations, measured via caregiver behavior and/or attitudes, was connected to sociomoral outcomes in the preschool years and (retrospectively, at least) in adulthood. Importantly, our tests of these relations were largely conducted in the context of controlling for maternal responsivity, meaning that the relations we found were not a function of generally sensitive and responsive care, but related specifically to touch (although maternal affectionate touch in the first months of life predicts maternal responsivity later; see Field et al., 1994). Consistency across studies suggests that the connection between maternal touch orientation and moral development might emerge both through sociomoral capacities, such as empathy and behavioral regulation, as well as through children’s developing moral orientations. Maternal touch orientations might also relate to the appearance or emergence of psychopathology, which appears to have implications for the relation between maternal touch orientations and sociomoral development.

#### Moral capacities

In these studies, maternal touch orientations related to several sociomoral capacities, such as empathy and behavioral regulation. For example, empathy related to both positive and negative maternal touch attitudes in study 1, and to both positive and negative touch behavior during childhood in study 3. The consistency of these findings connecting empathy and maternal touch orientations is supported by the literature showing that touch increases attention, compliance, and even generosity toward others in adults (Gueguen & Fischer-Lokou, 2003; Joule & Gueguen, 2003).

Our findings also suggest a significant role for the relation between maternal touch orientations and behavioral regulation. Connections between maternal touch orientations and inhibitory control/behavioral regulation emerged in studies 1 and 2. In particular, the ability to control one’s impulses seemed correlated with concurrent positive, affectionate touch, lack of negative touch, and touch attitudes. These results suggest an important role for touch, particularly caregivers’ avoidance of negative touch, in the development of regulatory systems with implications for interpersonal interaction. This idea is consistent with findings that experiencing positive touch increases adaptive vagus nerve activity and oxytocin release while decreasing cortisol (see Field, 2010, for a review), decreasing social anxiety, and instead allowing openness in social interactions (Porges, 2011).

#### Moral orientation

Studies 1 and 3, despite their differential focus on child versus adult outcomes, showed consistent findings with respect to the relations between touch and moral orientation. In both studies, moral orientations associated with social engagement were positively connected to affectionate touch and lack of punishing touch, with opposite relations for moral orientations associated with safety or self-protection (social opposition and withdrawal). The measures of social engagement and cooperation in study 2, although behavioral manifestations of engagement with others rather than measures of moral orientation per se, also showed significant positive relationships with a lack of negative touch in late toddlerhood. Generally, experiencing positive touch appeared to facilitate and encourage engagement with others, whereas experiencing punishing touch did not.

#### Psychopathology

Studies 1 and 3 provided evidence of a link between early caregiver touch orientations and outcomes related to psychopathology. The negative correlations between depression and anxiety and maternal attitudes toward positive and negative touch in study 1 dovetail with the correlations between retrospective reports of childhood touch and scores on internalizing pathology in study 3. Mental health scores were also a significant component of the mediation models predicting moral orientation in adulthood. These findings suggest that part of the pathway that connects early caregiver touch to individuals’ moral functioning is through the direct and indirect effects of touch on mental health and wellbeing.

Why might touch matter for mental health? “An absence of positive social interactions early in life, especially those involving physical contact with caregivers, helps set a low threshold for activating the amygdala in response to potential threats that may persist throughout the lifespan” (Ochsner & Gross, 2007, p. 103). Limited touch in early life leads to an underdevelopment of serotonin receptors, endogenous opioids, and oxytocin (Kalin, 1993; Meinischmidt & Heim, 2007), whereas under normal conditions, physical touch activates calming hormones such as oxytocin (Kramer, Cushing, & Carter, 2003; Carter, 2003; Liu et al., 1997). Oxytocin usually rises in typically developing children when touched by parents, but not in Romanian orphans adopted after several years in an orphanage. One interpretation of these findings is that the absence of affectionate touch experienced by orphans results in atypical development of physiological systems during a sensitive period (Fries, Ziegler, Kurian, Jacoris, & Pollak, 2005). Although the specific implications of physiological differences between typically developing children and Romanian orphans is unclear, physiology might well play a significant role in social challenges sometimes experienced by the latter.

### Positive versus negative touch

We explored the different effects that positive versus negative touch attitudes and behavior had on child outcomes. Though positive versus negative touch variables showed influences on sociomoral outcomes, the patterns were not consistent across studies. For example, taken alone, longitudinal data in study 2 results suggest that avoiding negative touch is more important than providing positive touch. In studies 1 and 3, significant, opposite patterns were found for positive and negative touch orientations. Perhaps the different findings for study 2 vis-a-vis the other studies were owing to method. In studies 1 and 3, the same respondent provided information both on attitudes/behaviors and on outcomes. In study 2, maternal reports of attitudes and parenting were compared with observations of child behavior and were not subject to the same level of participant bias as the other studies. Future research could usefully examine families in which corporal punishment is absent, but a range of positive touch is present. Variations in child behavioral outcomes as a function of individual differences in maternal positive touch would corroborate the results of the other studies suggesting that increases in positive touch are associated with increases in sociomoral behavior independently of avoidance of negative touch.

### Touch attitudes and touch behavior

We explored whether there were differential relations between maternal touch *attitudes* and touch *behavior* in regards to sociomoral outcomes in study 2. The results between touch behavior and attitudes overlapped quite a bit, but there were more correlations for negative than for positive variables. However, comparisons of attitudes and behaviors were difficult in study 2 because of the timing differences in the measurement of attitudes and behavior and because attitudes were measured only once. Nevertheless, negative touch attitudes (i.e., rejection of punishment) were the only touch variable that related to child internalizing behaviors (and only at age 2). The other correlations with attitudes also show up in relation to touch behaviors at 30 months, suggesting that touch behavior might continue to interact with touch attitudes. Further research could examine whether relations among attitudes, behaviors, and child outcomes are best explained by linear or mediational models. Quite possibly, the effects of particular parenting behaviors on child outcomes are mediated by parents’ attitudes.

## Limitations

One of the strengths of the manuscript is that we used different data sets and ages to examine the primary question, does touch influence sociomoral wellbeing? One of the weaknesses is that we do not have experimental data. It would be unethical to assign children to high/low touch environments, so addressing the question must rely on animal experiments and correlational studies with human beings.

The greatest limitation to this research is that early development is highly dynamic, with systems and subsystems developing on a maturational schedule and the relational environmental effects shifting in interaction with the individual’s course of development. This process-relational and relational developmental systems approach emphasizes becoming and change as fundamental categories for a nonlinear, coconstructive, layered process with multiple co-acting factors (Overton & Molenaar, 2015). Here, we only took static, impressionistic measurements of what is a dynamic enterprise. However, it would be difficult to set up and fund a way to measure the ongoing effects of dynamic relationships.

The research presented here had additional limitations. We used primarily maternal reports of behavior and retrospective reports of experience. There was limited examination of touch measured through behavioral observation. The self-reported behavior pertaining to touch may be impacted by socially desirable responding (reporting more affectionate touch and less punitive touch). Our designs, age groups, and measures were not identical from one study to the next, which was useful on the one hand for providing converging evidence with multiple approaches, but makes comparing across studies a little more difficult. Also, some variables had very limited variance suggesting that further sampling must be done to evaluate if the same relationships are sustained with a larger range in responses.

## Conclusions

Recall that we use as a baseline the evolved developmental niche, the set of practices that humans evolved to care for our young. Our findings suggest that moving away from the EDN provision in relation to positive touch (and lack of negative touch) is a risk factor for illbeing and poorer social and moral outcomes. Touch-related behaviors consistent with the EDN appear to communicate support for the child’s developing psychosocial neurobiology.

The findings presented here suggest an important role for early experiences of touch in sociomoral development. These findings suggest that positive touch should be encouraged among those who care for children, including families and childcare workers. At the same time, negative touch should be avoided. These findings reinforce what has already been suggested by others, that touch is fundamental for proper human development (e.g., Montagu, 1971).

Although the differential patterns of association between positive and negative touch and mechanisms by which touch affects social interaction and the development of morality require clarification in future research, the associations between touch and such outcomes emerged even when controlling for generally sensitive and responsive care. Touch thus might make unique contributions to the development of feelings of social connectedness and moral obligation. Quite possibly, the extent to which we see ourselves as engaged with and responsible for the health and wellbeing of others might be partly owing to the physical affection and/or corporal punishment we have experienced, particularly in early life.

## Future directions

Our findings suggest an avenue for future research. The links between touch and moral capacities are likely based in the functioning of physiological systems, as supported both by previous research using both humans (Field, 2010) and animals (Field, 1995) and the connections we found between touch and various measures of behavioral regulation. Direct measurements of physiological systems could enhance the study of how caregiver touch is connected to processes such as empathy and perspective-taking. For example, oxytocin, a hormone associated with feelings of calm (Feldman, 2012; Parker et al., 2014), elicited by affectionate touch (Uvnas-Moberg, 1997), promotes processes of social engagement by lowering stress-related defenses. Measures of both parent and child touch and the effects on multiple physiological systems would be revelatory. Already moving in this direction, Feldman (2007) has demonstrated that parent-child bio-social synchrony depends on physiological mechanisms, such as oxytocin release, which in turn foster self-regulation and capacities for empathy in childhood and adolescence.

## Data Availability

Data for studies 1 and 3 are available via request from the first author. Data for study 2 are available through the National Data Archive for Child Abuse and Neglect at Cornell University, Ithaca, New York, by requesting data set 140.

## Abbreviations

AL: Alabama
CRQ: Close Relationship Questionnaire
CTEM: Child Triune Ethics Measure
DC: District of Colombia
EDN: Evolved developmental niche
EDNH: Evolved developmental niche history
HOME: Home Observation for the Measurement of the Environment
IDAS: Inventory of Depression and Anxiety Symptoms
IN: Indiana
ITSEA: Infant-Toddler Social and Emotional Assessment
K: 1000
KS: Kansas
MO: Missouri
PFT: Parenting for the First Time project
TEM: Triune ethics meta-theory
USA: United States of America

## Footnotes

1 A caveat of this second data analysis is that the reliabilities of the touch behavior variables were low and thus the corresponding observed correlations reported in Table 3 were attenuated (Spearman, 1904).

2 To evaluate the overall mediation effect for each mediation model, we conducted the joint test of significance. Specifically, we tested the null hypothesis: *H*0 : *b*1 × *b*2 × *b*3 × *b*4 = 0. To this end, we obtained the 95% bootstrap bias corrected confidence intervals to estimate *b*1 × *b*2 × *b*3 × *b*4. If the 95% bootstrap bias corrected confidence interval of the overall indirect effect *b*1 × *b*2 × *b*3 × *b*4 does not include 0, we conclude that the overall mediation effect is significantly different from 0 at the 0.05 significance level. With a significant mediation effect, if *c*’ is not significantly different from 0, it is called a complete mediation; otherwise, it is a partial mediation (MacKinnon, 2008). This approach for testing indirect effects has been recommended due to its good performance in terms of both type I error rates and statistical power via simulation studies (MacKinnon, Lockwood, Hoffman, West, & Sheets, 2002; Hayes & Scharkow, 2013). All the mediation models were fitted in Mplus.
